# Diameter Class-Dependent Species-Specific Tree–Soil Feedback Linked to Soil Quality Between *Cunninghamia lanceolata* (Lamb.) Hook. and *Quercus fabri* Hance in Subtropical Forests

**DOI:** 10.3390/plants15030402

**Published:** 2026-01-28

**Authors:** Gang Lei, Yang Yang, Wenting Li, Tian Chen, Lianghua Qi

**Affiliations:** 1 International Center for Bamboo and Rattan, Beijing 100102, China; leigang@icbr.ac.cn (G.L.); yangyang@icbr.ac.cn (Y.Y.); liwenting@icbr.ac.cn (W.L.); 2 Sanya Research Base, International Center for Bamboo and Rattan, Sanya 572000, China; 3 College of Life and Environmental Sciences, Huangshan University, Huangshan 245041, China; chentianenci@caf.ac.cn

**Keywords:** tree–soil feedback, diameter classes, species-specific, soil quality index

## Abstract

The coupling between tree biomass and soil microhabitats is central to subtropical forest soil functioning, yet species- and stage-specific tree–soil interactions remain understudied. This study quantified these interactions in two dominant species—*Cunninghamia lanceolata* (Lamb.) Hook. (*C. lanceolata*) and *Quercus fabri* Hance (*Q. fabri*)—across five diameter at breast height (DBH) classes (5–10, 10–15, 15–20, 20–25, 25–30 cm). Soil quality was characterized via the Soil Quality Index (SQI) based on 16 physicochemical and enzyme activity parameters, while random forest models identified biomass importance. Soil properties and enzyme activities varied with diameter class (*p* < 0.05): *C. lanceolata* showed a unimodal pattern (minimum at 15–20 cm DBH), whereas *Q. fabri* increased consistently (peaking at 20–30 cm DBH). The diameter class × species interaction significantly influenced SQI (*p* < 0.01): *Q. fabri* showed higher SQI than *C. lanceolata* at larger DBH, and vice versa at smaller DBH. Aboveground biomass dominated SQI variation in *C. lanceolata* (weight = 0.57), whereas belowground biomass dominated in *Q. fabri* (weight = 0.52; model *R*^2^ > 0.75). These findings demonstrate that DBH size and species identity jointly shape soil microenvironments, providing a mechanistic basis for informed subtropical forest management.

## 1. Introduction

In forest ecosystems, trees do not exist in isolation but form dynamic co-evolutionary complexes with tree-influenced soil microhabitats [1,2]. Tree biomass, a key indicator of forest productivity and carbon sequestration, accumulates with growth traits such as diameter at breast height (DBH) and tree height, and is integral to this co-evolutionary process [3]. Traditional studies largely focused on unidirectional effects—either soil influencing trees or vice versa—whereas modern ecological theory highlights continuous bidirectional feedback between the two [4]. Soil physicochemical properties (e.g., nutrient pools, pH) and biochemical activities (e.g., enzyme activities) provide conditions for tree establishment, growth, and competition, acting as a filtering mechanism [5,6,7,8]. In turn, trees reshape soil microhabitats via canopy structure, litter inputs, and root exudates, engineering soil properties [9]. This bidirectional feedback drives spatial heterogeneity in belowground processes. Feedback intensity and direction exhibit species specificity and stage dependency [10,11,12], suggesting tree species with distinct life history and resource use strategies have fundamentally different soil interaction mechanisms [13]. For example, across forest ecosystems in Hunan Province, vegetation type determined a distinct soil quality rank: shrubland > bamboo forest > broadleaved forest > conifer–broadleaved mixed forest > coniferous forest, attributed to intensifying stand competition [14]. In subtropical secondary forest restoration, evergreen coniferous, conifer–broadleaved mixed, and deciduous broadleaved stands differed in soil enzyme activities and microbial nutrient limitation: evergreens eased microbial P limitation, whereas deciduous broadleaved trees eased microbial C limitation, and pioneer/mid-successional deciduous species best balanced microbial nutrient demands [15]. As trees develop and DBH increases, dynamic shifts in biomass, morphology, and resource modification capacity alter tree–soil feedback [1]. Investigating the relationships among tree species, size, and soil microhabitat relationships at the individual level is critical for understanding forest co-evolutionary mechanisms. However, current vegetation–soil interaction research focuses on stand- or regional-scale analyses, homogenizing individuals of varying sizes and neglecting feedback driven by species identity and DBH class at the individual tree level [16,17,18]. Averaging such fine-scale variation risks obscuring key ecological processes. For functionally divergent, long-term coexisting tree species, species-specific feedback with soil microhabitats via abiotic pathways across growth stages remain critical research gaps.

Building on these gaps, this study selected two subtropical Chinese tree species—*Cunninghamia lanceolata* (Lamb.) Hook. (*C. lanceolata*) and *Quercus fabri* Hance (*Q. fabri*)—as focal taxa. *C. lanceolata* is a major timber species in China with high economic value and strong ecological adaptability [19]; *Q. fabri*—with an extensive root system and distinct litter decomposition traits—contributes significantly to ecosystem stability [20]. Adopting an individual-tree framework, this study compared tree-influenced soil microenvironment characteristics across diameter classes and quantified soil quality via the Soil Quality Index (SQI), integrating soil physicochemical properties (bulk density, pH, capillary/non-capillary porosity), nutrients (organic matter, total/available nitrogen and phosphorus), and enzyme activities (urease, peroxidase, acid phosphatase, and other hydrolases and oxidases). This study aimed to clarify how tree size and species identity jointly regulate soil microenvironments and biomass–soil quality coupling in subtropical plantations, thereby informing precision forest management. To address this overarching goal, three independent hypotheses were tested, corresponding to key questions about size effects, species effects, and biomass–soil coupling: (1) Soil microenvironments differ among conspecific individuals across diameter classes. (2) At equivalent sizes, *C. lanceolata* and *Q. fabri* form distinct soil microenvironments. (3) Species differ in biomass–SQI relationships.

## 2. Results

### 2.1. Analysis of Growth Characteristics of Forests Across Different Size Classes

Table 1 shows distinct population dynamics for *C. lanceolata* and *Q. fabri* across diameter classes. *C. lanceolata* exhibited a unimodal distribution: stem density peaked in Class III at 42 trees, then declined to 8 trees by Class V. *Q. fabri* showed a similar pattern, peaking at 30 trees in Class III and decreasing to 16 trees in Class V. These structural differences suggest divergent regeneration and survival strategies between the two species. Intra-class variability was low for *C. lanceolata*, with coefficients of variation (CV) under 50% for most growth parameters—diameter at breast height (DBH), tree height, and above- and belowground biomass—except belowground biomass in Class I, which reached 63.6%. Between-class variation was high for biomass, with CVs of 128.2% and 97.4% for aboveground and belowground components, respectively. This high between-class variation underscores strong size-dependent effects on biomass accumulation. For *Q. fabri,* intra-class CVs were also below 50%, but between-class CVs were markedly higher (495.8% for aboveground biomass and 405.7% for belowground biomass), indicating greater size-dependent heterogeneity than that in *C. lanceolata*. Mean DBH for *C. lanceolata* increased from 2.1 to 17.8 cm between Class I and V, and aboveground biomass rose from 0.7 to 213.5 kg. *Q. fabri* exhibited a similar DBH increase (1.9 to 20.4 cm) but higher biomass accumulation (0.8 to 285.3 kg), confirming positive size-dependent growth.

### 2.2. Soil Factor Characteristics Across Size Classes

**Figure 1:**
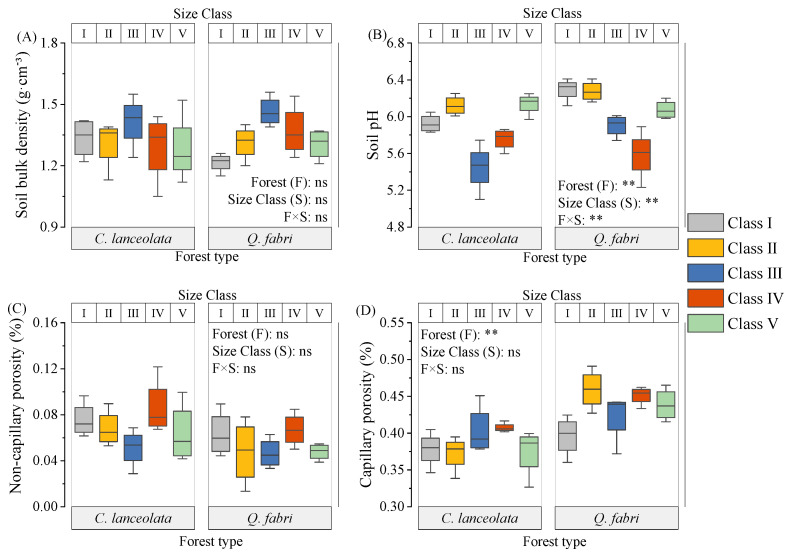
Soil physical and chemical properties across diameter classes in *C. lanceolata* and *Q. fabri* forests. Note: (**A**–**D**) show bulk density, pH, non-capillary porosity, and capillary porosity (means ± SE). Symbols *, **, and ns denote the significance of two-way ANOVA (forest type × size class) interactions: *p* < 0.05, *p* < 0.01, and non-significant, respectively. *Cunninghamia lanceolata* (Lamb.) Hook. (*C. lanceolata*) and *Quercus fabri* Hance (*Q. fabri*) were the two tree species investigated in this study.

**Figure 2:**
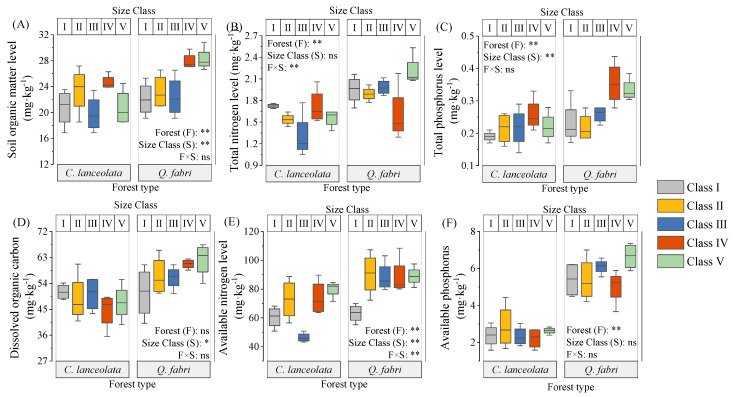
Differences in soil nutrient indicators across diameter classes in *C. lanceolata* and *Q. fabri* forests. Note: (**A**–**F**) represent soil organic matter, total nitrogen, total phosphorus, dissolved organic carbon, available nitrogen, and available phosphorus, respectively. Symbols *, **, and ns denote the significance of two-way ANOVA (forest type × size class) interactions: *p* < 0.05, *p* < 0.01, and non-significant, respectively. *Cunninghamia lanceolata* (Lamb.) Hook. (*C. lanceolata*) and *Quercus fabri* Hance (*Q. fabri*) were the two tree species investigated in this study.

**Figure 3:**
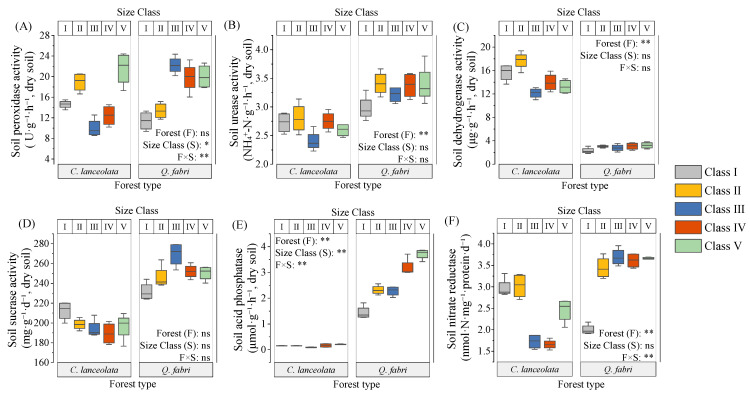
Differences in soil enzyme activities across diameter classes in *C. lanceolata* and *Q. fabri* forests. Note: (**A**–**F**) represent soil peroxidase, urease, dehydrogenase, sucrase, acid phosphatase, and nitrate reductase activities, respectively. Symbols *, **, and ns denote the significance of two-way ANOVA (forest type × size class) interactions: *p* < 0.05, *p* < 0.01, and non-significant, respectively. *Cunninghamia lanceolata* (Lamb.) Hook. (*C. lanceolata*) and *Quercus fabri* Hance (*Q. fabri*) were the two tree species investigated in this study.

**Figure 4:**
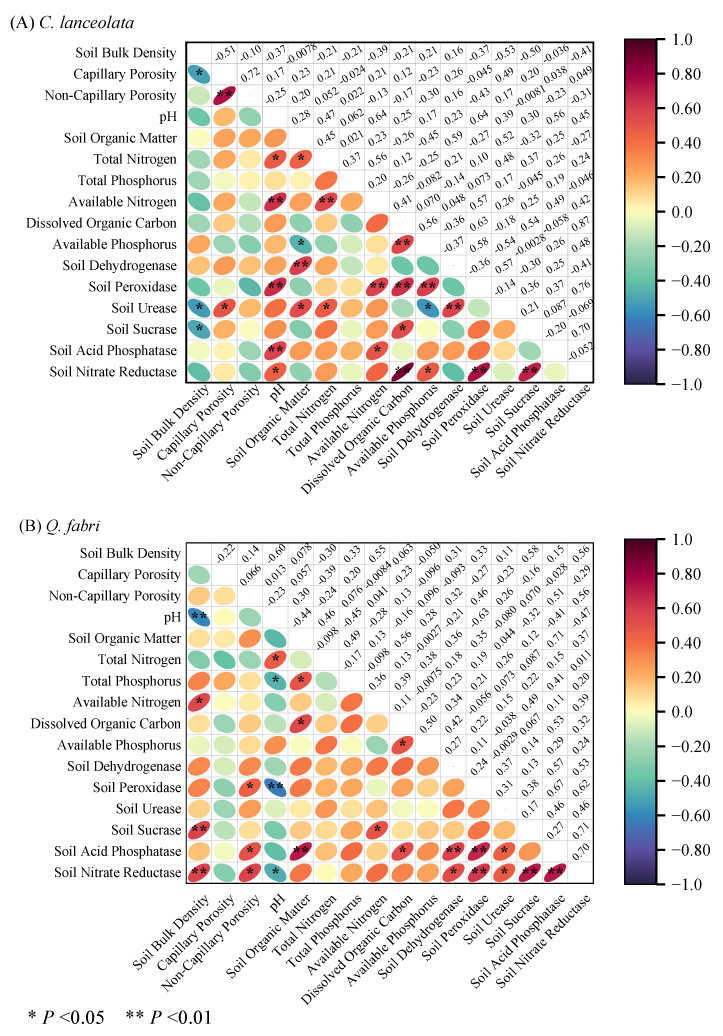
Correlation analysis of soil factor characteristics in *C. lanceolata* and *Q. fabri* forests. Note: (**A**,**B**) represent correlation networks for *C. lanceolata* and *Q. fabri* forests, respectively. ** and * denote significance at *p* < 0.01 and *p* < 0.05, respectively. *Cunninghamia lanceolata* (Lamb.) Hook. (*C. lanceolata*) and *Quercus fabri* Hance (*Q. fabri*) were the two tree species investigated in this study.

In *C. lanceolata* forests, bulk density and porosity did not vary among diameter classes, whereas Class III pH was lower than that of other classes (*p* < 0.05, Figure 1 and Table S2). Soil organic matter, total nitrogen, available nitrogen, peroxidase, and urease showed unimodal patterns with minima in Class III. These minima likely reflect intense nutrient uptake during peak growth in Class III. Dissolved organic carbon and available phosphorus were invariant among classes (Figure 2 and Figure 3). Bulk density correlated negatively with urease, sucrase, and capillary porosity, while pH correlated positively with total nitrogen and most enzymes (Figure 4A). Soil organic matter correlated positively with total nitrogen and most enzymes but negatively with available P (Figure 4A). These relationships indicate tight coupling between soil physical properties, nutrient pools, and microbial functions in *C. lanceolata* stands.

In *Q. fabri* forests, bulk density and pH were unimodal: Class V bulk density exceeded Class I (*p* < 0.05) and Class V pH was lower than Class IV (*p* < 0.05). Non-capillary porosity remained stable, whereas Class I capillary porosity was lower than other classes (*p* < 0.05, Figure 1 and Table S2). Soil organic matter, total phosphorus, dissolved organic carbon and available nitrogen increased to peaks in Classes IV–V, while total N was unimodal with the highest value in Class V (Figure 2). Available phosphorus was lowest in Class IV (Figure 2 and Table S4). Bulk density correlated negatively with pH but positively with available nitrogen and most enzymes, while pH correlated positively with total nitrogen but negatively with total P and most enzymes (Figure 4B). Most enzyme activities were positively correlated (Figure 4B).

Interspecific comparisons revealed that forest type affected pH, capillary porosity, most nutrients, and urease and dehydrogenase activities (Figure 1, Figure 2 and Figure 3). Diameter class influenced pH, soil organic matter, total phosphorus, peroxidase and acid phosphatase (Figure 1, Figure 2 and Figure 3). Interactive effects were observed for pH, total nitrogen, total phosphorus and most enzymes (Figure 1, Figure 2 and Figure 3). Across most classes, *C. lanceolata* forests had lower bulk density, pH, nutrients, sucrase and acid phosphatase than *Q. fabri* forests (Table S3). Conversely, dehydrogenase, Class II peroxidase and Class I nitrate reductase were lower in *Q. fabri* forests (Table S3).

### 2.3. Soil Microhabitat Characteristics of Forests Across Different Size Classes

Principal component analysis (PCA) revealed distinct soil feature structures between *C. lanceolata* and *Q. fabri* forests. In *C. lanceolata*, five key components explained 81.2% of the total variance (Table 2). PC1 accounted for 27.5% of the variance and correlated strongly with pH, available nitrogen, and nitrate reductase activity, reflecting soil acidity and nitrogen availability. PC2 explained 24.5% of the variance and correlated with soil organic matter and urease activity, indicating organic matter supply capacity. PC3–PC5 each contributed <10% and were associated with bulk density, porosity, and phosphorus availability. These components indicate that *C. lanceolata* soils are primarily governed by pH–nitrogen interactions. In *Q. fabri,* six components explained 81.7% of the variance (Table 3). PC1 explained 32.0% of the variance and correlated with bulk density, acid phosphatase, and nitrate reductase activity, indicating soil structural properties and enzymatic activity. PC2 accounted for 15.1% of the variance and correlated with pH and available phosphorus, reflecting phosphorus availability. PC3–PC6 each contributed <10% and were linked to nitrogen cycling and porosity. In contrast, *Q. fabri* soils are dominated by bulk density–phosphorus relationships. These distinct PCA structures provide a multivariate foundation for understanding species-specific soil modification strategies.

**Figure 5:**
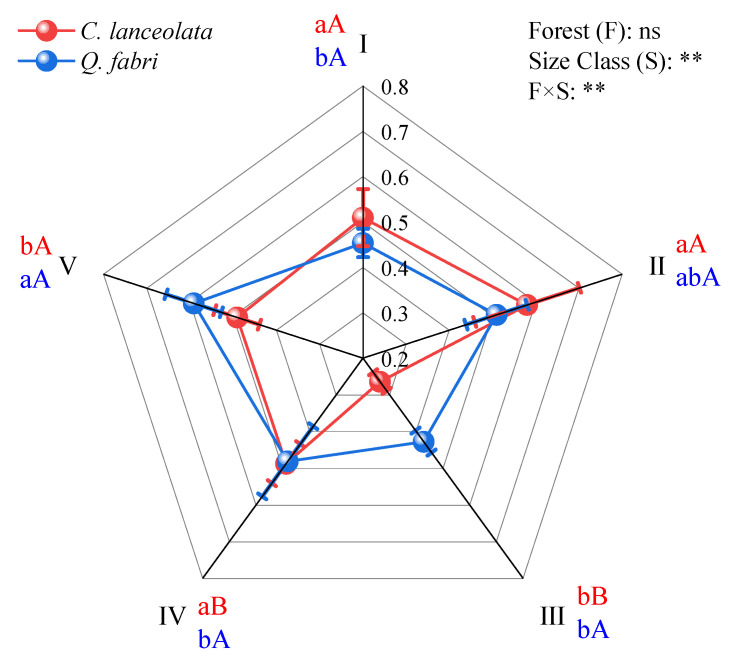
Differences in soil quality index across diameter classes in *C. lanceolata* and *Q. fabri* forests. Note: Within species, different lowercase letters indicate significant differences among DBH classes (*p* < 0.05); between species at the same DBH class, different uppercase letters indicate significant differences (*p* < 0.05). Symbols *, **, and ns denote the significance of two-way ANOVA (forest type × size class) interactions: *p* < 0.05, *p* < 0.01, and non-significant, respectively. *Cunninghamia lanceolata* (Lamb.) Hook. (*C. lanceolata*) and *Quercus fabri* Hance (*Q. fabri*) were the two tree species investigated in this study.

PCA-derived SQI increased with diameter class in both forest types. In *C. lanceolata*, SQI rose from Class II to V, peaking in Class V (*p* < 0.05, Figure 5). Similarly, *Q. fabri* exhibited optimal soil microhabitats in Class V, with values higher than Class II (*p* < 0.05, Figure 5). Forest type did not affect SQI, whereas diameter class and the forest type × diameter class interaction had significant effects (*p* < 0.001, Figure 5). Specifically, *Q. fabri* Class V soils had higher quality than *C. lanceolata* Class V soils, whereas *C. lanceolata* Class I soils had slightly higher SQI than *Q. fabri* Class I soils. These results demonstrate that larger diameter classes support superior soil microhabitats.

**Figure 6:**
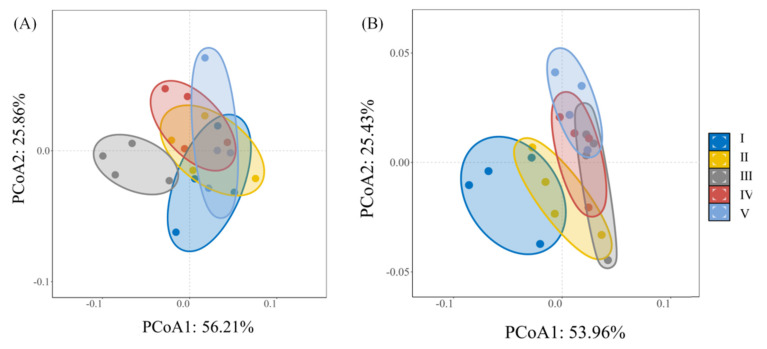
Principal coordinate analysis (PCoA) of soil factors in *Cunninghamia lanceolata* (Lamb.) Hook. (**A**) and *Quercus fabri* Hance (**B**) forest.

Principal coordinate analysis (PCoA) of *C. lanceolata* soils showed that PCoA1 and PCoA2 explained 56.2% and 25.9% of the variance, respectively (82.1% cumulative; Figure 6A). These axes captured core differences in soil properties, with Class III samples clearly separated from other classes. This separation indicates that Class III represents a distinct soil microenvironment phase in *C. lanceolata* development. In *Q. fabri* forests, PCoA1 and PCoA2 explained 54.0% and 25.4% of the variance (79.4% cumulative; Figure 6B), and Class I samples were distinctly separated from other classes. Early-stage separation in *Q. fabri* suggests that this species establishes distinct soil conditions from the sapling stage. Thus, diameter class drove soil property differentiation in both forest types, but with different thresholds: *C. lanceolata* showed a mid-stage transition (Class III), whereas *Q. fabri* exhibited early differentiation followed by stabilization (Class I).

### 2.4. Differential Effects of Forest Biomass Types on Soil Quality Index

**Figure 7:**
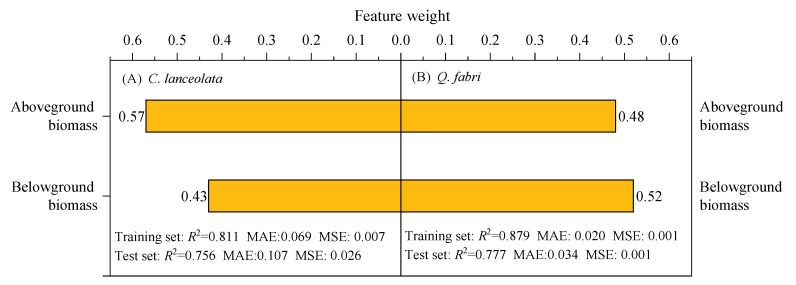
Feature weights of aboveground and belowground biomass on soil quality index from random forest analysis. Note: (**A**) *Cunninghamia lanceolata* (Lamb.) Hook. and (**B**) *Quercus fabri*. Hance MAE = mean absolute error; MSE = mean squared error.

Using random forest models with SQI as the dependent variable, the importance of aboveground and belowground biomass in predicting soil quality for *C. lanceolata* and *Q. fabri* was quantified (Figure 7). The two species exhibited contrasting biomass–soil quality relationships: aboveground biomass was the primary driver for *C. lanceolata* (feature weight: 57% of variance explained), whereas belowground biomass dominated for *Q. fabri* (feature weight: 52%). Model performance was robust, with training *R*^2^ values of 0.811 and 0.879 for *C. lanceolata* and *Q. fabri*, respectively, and testing *R*^2^ > 0.75 for both species (low MAE and MSE). These findings confirm that random forest models effectively capture nonlinear biomass–soil quality associations, revealing species-specific regulation: *C. lanceolata* relies primarily on aboveground processes, while *Q. fabri* depends more on belowground processes.

## 3. Discussion

### 3.1. Species-Specific Diameter Class-Dependent Tree–Soil Interactions

This study documents differences in diameter class structure between *C. lanceolata* and *Q. fabri*, relating to divergent ecological strategies associated with species-specific niche differentiation. *C. lanceolata* had greater abundance in Class I (21% of the total) and its abundance peaked at Class III (35% of the total), whereas *Q. fabri* showed a different pattern at Class I and Class III (12.5% and 25% of the total, respectively). Table 1 visualizes these patterns, showing *C. lanceolata*’s higher Class I recruitment and Class III dominance versus *Q. fabri*’s lower initial recruitment. This pattern in *C. lanceolata* is consistent with cohort-driven regeneration following disturbance. Such post-disturbance regeneration directly impacts soil legacies through rapid litter and root inputs. In contrast, *Q. fabri* displayed a unimodal diameter class distribution, with mean DBH in Class V larger than that of *C. lanceolata* (20.37 vs. 17.81 cm). This pattern is associated with delayed maturation under closed-canopy conditions (Table 1). Delayed maturation allows *Q. fabri* to accumulate belowground biomass, intensifying root–soil interactions. Such size-dependent structural divergence directly impacts soil development trajectories, as larger trees intensify belowground resource partitioning. These diameter class distribution patterns are consistent with previously reported interspecific differences in shade tolerance and light acquisition strategies [21,22,23].

Soil physicochemical properties and enzyme activities varied significantly across diameter classes within each species (Figure 1, Figure 2 and Figure 3), supporting Hypothesis 1. These variations underscore that size-dependent growth stages actively engineer soil conditions rather than passively reflecting them. For *C. lanceolata*, soil organic matter (SOM), total nitrogen (TN), and most enzyme activities followed a decline-then-rise trajectory, with minima at Class III—the stage of most rapid aboveground growth (Figure 2 and Figure 3, Tables S3 and S4). These minima in Class III reflect peak nutrient demand during active growth. This pattern aligns with the growth–nutrient demand–litter feedback mechanism [24]: intense nutrient uptake during stem elongation is associated with transient soil nutrient depletion [25], which is subsequently mitigated by increased litter input as trees mature. These distribution patterns reveal species-specific regeneration strategies that differentially influence soil legacy development. Table 1 corroborates this interpretation, showing a threefold biomass increase from Class III to V that drives organic matter recovery. Aboveground biomass nearly tripled from Class III to Class V (from ~68.2 kg to ~213.49 kg; Table 1), linked to recovery of SOM through decomposition processes [19]. This biomass-driven organic matter recovery is visually evident in Figure 2A, where SOM rises after Class III. Concurrently, soils in Class III had a significantly lower pH (Figure 2B), a pattern potentially linked to exudation of organic acids by roots—an established strategy for nutrient mobilization [26,27]. These findings collectively demonstrate that *C. lanceolata* actively modifies its soil microenvironment through size-dependent resource allocation. In contrast, *Q. fabri* displayed distinct soil dynamics: bulk density initially increased then declined, pH decreased before rising again, and key soil nutrients generally increased, with peak values observed in Classes IV–V (Figure 1 and Figure 2, Tables S2 and S4). *Q. fabri* shows a different temporal pattern than *C. lanceolata*. These trends align with characteristics of its broadleaf litter, including a lower C:N ratio [28,29]—a trait associated with enhanced decomposability and more efficient nutrient cycling [30]. PCoA revealed that *Q. fabri* in Class I formed a distinct cluster (Figure 6B). This pattern is indicative of early-stage tree-influenced soil microenvironment specialization. This differs from *P. sylvestris*, where microhabitats of juvenile trees are minimally influenced by adult individuals [31]. Such ontogenetic shifts have direct management implications: retaining large-diameter trees sustains soil legacies, while species selection should consider these divergent temporal dynamics.

The significant species × diameter class interaction effect on soil parameters (Figure 1, Figure 2 and Figure 3) provides empirical support for Hypothesis 2. This interaction effect demonstrates that species effects are size-dependent, not fixed, reinforcing the need for diameter class-based management. Cross-species comparisons at equivalent diameter classes showed that *C. lanceolata* stands had lower bulk density, pH, sucrase activity and acid phosphatase activity than *Q. fabri* stands (Figure 1), while dehydrogenase activity was lower in *Q. fabri* stands (Figure 3), reflecting fundamental differences in coniferous versus broadleaf litter and root traits. These trait-mediated mechanisms, illustrated by the contrasting soil chemistry in Figure 2, provide a mechanistic basis for species-specific fertilizer prescriptions. These differences relate to divergent litter chemistry: slow-decomposing needle litter of *C. lanceolata* is linked to long-term soil acidification [32], whereas labile leaf litter of *Q. fabri* associates with enhanced microbial activity. SQI analysis revealed no main effect of forest type, but a significant species × diameter class interaction (*p* < 0.01; Figure 5), which shows divergent SQI trajectories between the two species across diameter classes, confirming that species effects are size-dependent and reinforcing diameter class-based management. In Class V, *Q. fabri* stands had higher SQI than *C. lanceolata* stands, while *C. lanceolata* had slightly higher SQI than *Q. fabri* in Class I (Figure 5). This observation is consistent with Obade and Lal [33], who asserted that soil quality is shaped by biological traits and environmental context, and it highlights diameter class as a regulatory mediator of tree–soil interactions. These SQI patterns provide a quantitative basis for identifying low-quality stands: Class III *C. lanceolata* and Class I *Q. fabri* represent priority intervention zones. These findings are supported by additional data: the ordination plot in Figure 6A shows distinct clustering of Class III *C. lanceolata* samples, indicating a unique soil condition at this diameter class. Similarly, Class I *Q. fabri* samples formed a separate cluster (Figure 6B), suggesting early divergence in soil properties.

### 3.2. Diameter Class-Mediated Species Differences in Biomass–Soil Quality Coupling

Random forest analysis supported Hypothesis 3, delineating interspecific differences in biomass-driven SQI variation (Figure 7). The tree–soil relationship is not passive but dynamic and species-specific, with biomass and soil conditions mutually shaping each other. This feedback specificity reflects species-specific resource allocation and functional traits. For *C. lanceolata*, aboveground biomass weight was 0.57 and belowground biomass 0.43 in explaining SQI variation (Figure 7A). This aligns with coniferous strategies: *C. lanceolata* prioritizes vertical growth with a high shoot-to-root ratio [23]. It allocates more resources to xylem formation to compensate for tracheids’ low water transport efficiency, with leaves of low N/P and high C content—emphasizing structural investment over rapid nutrient turnover [34]. Global conifer tree-ring data further confirm a widespread “fast growth–drought tolerance” trade-off [35]. This trade-off is embodied in the conservative growth strategy of *C. lanceolata*, which enhances environmental adaptability via structural resource investment. Conifer–broadleaf mixed forest studies support this stable growth strategy: *C. lanceolata*’s self-thinning trajectory matches that of pure stands, remaining within core thresholds without mixed-environment interference [36]. Higher aboveground biomass correlates with greater litter input, which indirectly modifies soil quality via decomposition, potentially forming a positive feedback loop.

*Q. fabri* showed the opposite pattern: belowground biomass contributed 52% to SQI variation versus 48% for aboveground biomass (Figure 7B). This aligns with broadleaf root traits: *Q. fabri* has a high fine root turnover rate, accelerating N/P release during decomposition [37]. Additionally, root-secreted organic acids convert soil P from stable to active forms via acidolysis, significantly improving P availability [38]. These processes form a dominant “belowground biomass–root processes–soil quality” pathway, consistent with the findings of Tedersoo et al. [39] and Norris et al. [40], who concluded that root–microbe interactions govern nutrient cycling in broadleaf forests. Furthermore, *Q. fabri* soil bacteria exhibit greater drought tolerance, while moderate shading elevates soil fungal diversity, strengthening belowground nutrient cycling stability [23]. These interspecific differences align with the “fast–slow continuum” framework: *C. lanceolata* (conifer) employs a “slow strategy” centered on structural investment and environmental adaptability, whereas *Q. fabri* (broadleaf) adopts a “resource-acquisitive strategy” focused on efficient nutrient turnover. This conclusion aligns with Shen et al. [14], who documented distinct biomass–soil coupling patterns across species with divergent life-history strategies in *P. massoniana* forests, further validating the generality of conifer-broadleaf feedback pathway differentiation reported herein.

Subtropical forest research indicates that conifers exhibit lower N resorption efficiency, greater seasonal fluctuations, and weaker disturbance resistance than broadleaves [41]. Plant–microbe interactions mediate these strategies: plants recruit specific soil microbes via chemical signals to enhance nutrient acquisition [42], while mycorrhizal fungi decompose organic matter and modulate nutrient fluxes through underground hyphal networks [39]. These microbial mechanisms, illustrated in our enzyme activity data (Figure 3), explain the functional differences observed between species. *C. lanceolata* relies on aboveground litter to regulate SQI; disturbances reduce litter quantity and quality and decomposition rate, impacting nutrient supplementation. In contrast, *Q. fabri* soil microenvironment feedback depends on root–microbe interactions, maintaining stability under moderate disturbances [43]. While random forest models revealed key patterns, they do not establish causal relationships in this study. This limitation is acknowledged in Figure 7, which shows correlation, not causation, between biomass and SQI. Future research should combine isotope tracing and time-series monitoring to verify biomass-soil feedback directionality, and incorporate mycorrhizal indicators to improve model explanatory power. This analytical framework (diameter class dynamics × species functional traits) advances mechanistic understanding of tree-soil interactions in subtropical forest ecosystems.

## 4. Materials and Methods

### 4.1. Study Area Overview

The study site (109°35′12″ E, 26°51′59″ N) lies in Moshao Village, Guangping Town, Huaihua, Hunan, China. Huitong County, in southwestern Hunan Province, lies within the mountainous region bordering the Yunnan–Guizhou Plateau–Jiangnan Hills transition zone the county is a core production area for *C. lanceolata*. The climate is subtropical monsoonal, with distinct wet–hot seasons. Mean annual temperature is 16.8 °C (January 4.8 °C, July 26.7 °C); 70% of the 1100–1400 mm annual precipitation falls during June–September, when monthly mean temperature exceeds 22 °C. Mean annual relative humidity is >80%. Soils are classified as loamy sand Luvisols (World Reference Base for Soil Resources, WRB) with a depth of approximately 40 cm. The specific study site is a hillside forestland with a southeast-facing aspect, a slope gradient of 40–57°, and an elevation range of 430–470 m.

### 4.2. Methodology

#### 4.2.1. Plot Selection and Field Survey

The selected forests were pure stands of *C. lanceolata* and *Q. fabri* established in 1999 following clear-cutting in the autumn of 1998. Since afforestation, stands have received uniform management without silvicultural interventions (e.g., thinning). The understory shrub layer is dominated by *Maesa japonica* (Thunb.) Moritzi., *Oreocnide frutescens* (Thunb.) Miq., *Schima superba* Gardn. et Champ., etc. The understory herbaceous layer mainly consists of *Woodwardia japonica* (L.f.) Sm., *Parathelypteris glanduligera* (Kunze) Ching., *Carex doniana* Spreng., *Lophatherum gracile* Brongn., etc. In August 2024, four 20 m × 20 m plots were established per species (eight plots total; Section 4.1). Plots were located in 25-year-old plantations with inter-plot spacing ≥20 m. Stand density was 750–800 stems·hm^−2^ for *C. lanceolata* and 550–650 stems·hm^−2^ for *Q. fabri*. Elevation was controlled during plot selection to ensure similar topography and community structure, minimizing external environmental interference. To ensure the reliability and representativeness of the data, all living trees with a diameter at breast height (DBH, measured at 1.3 m above ground) of ≥1 cm within each plot were identified and measured. To guarantee consistency and comparability across species, a proportional stratified random sampling method was employed, with 120 trees selected per species for detailed analysis. The specific stratification method is detailed in Section 4.2.2.

#### 4.2.2. Diameter Class Categorization and Biomass Calculation for Trees

Because DBH class reliably reflects tree developmental stage [44,45], all surveyed *C. lanceolata* and *Q. fabri* individuals were divided into five size classes: Class I: 1 cm < DBH ≤ 2.5 cm; Class II: 2.5 cm < DBH ≤ 5 cm; Class III: 5 cm < DBH ≤ 7.5 cm; Class IV: 7.5 cm < DBH ≤ 15 cm; Class V: 15 cm < DBH ≤ 30 cm [45]. The sampling ratio for each DBH class was determined according to its proportion in the total tree population of the corresponding stand. For example, if Class I accounted for 10% of the total population, then 12 trees from Class I were selected to be part of the 120 samples. This design ensured that the DBH class distribution of the selected sample was consistent with that of the entire plot. Compared to simple random sampling, this strategy effectively minimized potential biases and guaranteed that trees at different developmental stages were adequately and proportionally represented in the subsequent analysis. Biomass was calculated using allometric equations from the Chinese National Standard GB/T 43648–2024 [46]. The formula is as follows:

(1) Binary model for aboveground biomass:
  (1) $$M_{A}=a_{0}\times {\mathrm{DBH}}^{a_{1}}\times H^{a_{2}}$$
  where M_A_ = aboveground biomass (kg), DBH = diameter at breast height (cm), H = tree height (m), and a_0_, a_1_, a_2_ = species-specific parameters from GB/T 43648–2024 [46]. Parameter values are provided in Table 4.
(2) Binary model for belowground biomass:
  (2) $$M_{B}=b_{0}\times {\mathrm{DBH}}^{b_{1}}\times H^{b_{2}}$$
  where M_B_ = belowground biomass (kg), DBH = diameter at breast height (cm), H = tree height (m), and b_0_, b_1_, b_2_ = species-specific parameters from GB/T 43648–2024 [46]. Parameter values are provided in Table 5.
(3) Calculation Formula for Total Biomass:
  M_T_ = M_A_ + M_B_ (3)

#### 4.2.3. Soil Sample Collection and Soil Microhabitat Characterization

To ensure soil samples represented tree soil microhabitats, four soil blocks (10 cm × 10 cm × 20 cm) were excavated at the four cardinal points 1.5 m from each tree base and homogenized into one composite sample [1]. Prior to sampling, surface debris was removed. A subsample of each composite soil was passed through a 2 mm sieve, flash-frozen in liquid nitrogen, and stored at −20 °C for enzyme assays within one week. The remaining samples were transported to the laboratory, where they were air-dried, sieved (2 mm and 0.25 mm), and cleared of stones and root residues. Soil physicochemical properties—including bulk density (BD), capillary/non-capillary porosity, pH, soil organic matter (SOM), total nitrogen (TN), total phosphorus (TP), dissolved organic carbon (DOC), available nitrogen (AN), and available phosphorus (AP)—were analyzed following *Soil Agricultural Chemistry Analysis* (3rd ed.) [47]. Measured soil enzyme activities included peroxidase [48], dehydrogenase [49], urease [50], sucrase [51], acid phosphatase [52], and nitrate reductase [53].

Soil quality integrates physicochemical properties and ecosystem functionality. Therefore, the soil quality index (SQI) was used to evaluate soil microhabitats [1,54]. SQI was calculated via the Total Data Set (TDS) method in four steps [14]: (1) Averaging indicators, (2) Normalizing indicators, (3) Scoring indicators using correlation functions [55,56], and (4) Integrating scored values into a final index. For pH, given that all soils were acidic (pH < 7.0), a “higher-the-better” rule was applied reflecting proximity to neutrality (optimum 7.0). Parameters classified as “higher-the-better” (SOM, TN, TP, DOC, AN, AP) were scored accordingly. Enzymes involved in nutrient cycling (dehydrogenase, urease, sucrase, acid phosphatase) were also classified as “higher-the-better”. Peroxidase and nitrate reductase were classified as “lower-the-better” [57]. Specific equations are:

(4) $$Q=\frac{X-\mathrm{Xmin}}{\mathrm{Xmax}-\mathrm{Xmin}}$$

(5) $$Q=\frac{\mathrm{Xmax}-X}{\mathrm{Xmax}-\mathrm{Xmin}}$$

Here, Q denotes the membership degree (ranging from 0 to 1), X is the measured value of the indicator, X_min_ is the minimum value, and X_max_ is the maximum value of each indicator. Equation (4) was used for “higher-the-better” indicators, and Equation (5) for “lower-the-better” indicators. Principal component analysis (PCA) was subsequently performed on all soil indicators. Communal variances derived from PCA reflect the contribution of each indicator to the total variance, with larger values indicating greater importance [58]. Indicator weights were calculated from the PCA results using Equation (6), where *Ci* represents the absolute communal variance of indicator *i*.

(6) Wi=Ci∑Cii=1n

Finally, SQI under each forest stand was calculated based on the aforementioned Equation (7):

(7) SQI=∑WiQii=1n

Specifically, Q_i_ denotes the membership degree of indicator *i*, *n* represents the number of soil indicators, and *W_i_* is the weight of indicator *i* [59]. The SQI value was used to characterize soil microhabitat quality, with higher values indicating superior microhabitat conditions [1].

### 4.3. Statistical Analysis

Descriptive statistics were performed using SPSS Statistics 19.0 (IBM Corporation, Armonk, NY, USA) to characterize the distribution of stem density, diameter at breast height (DBH), tree height, aboveground biomass, and belowground biomass of *C. lanceolata* and *Q. fabri* within each diameter class. Levene’s test was used to assess homogeneity of variances, and Duncan’s honest significant difference (HSD) test was applied for post hoc multiple comparisons (*p* < 0.05). Pearson’s correlation analysis was conducted to evaluate relationships among soil physicochemical properties and enzyme activities (*p* < 0.05). Principal component analysis (PCA; see Section 4.2.3) was used to determine the communal variance of soil indicators for SQI calculation. Principal coordinate analysis (PCoA) was conducted to ordinate differences in soil quality among diameter classes. A random forest model was employed to quantify the explanatory power of tree biomass (aboveground and belowground) for SQI variation. Data visualization was performed using Origin 2024 (OriginLab Corporation, Northampton, MA, USA).

## 5. Conclusions

Tree biomass–soil coupling sustains soil functioning in subtropical forests but varies with diameter class and species. This study demonstrated that diameter class-dependent tree–soil interactions varied between *C. lanceolata* and *Q. fabri*, with within-species soil physicochemical properties and enzyme activities differing across diameter classes. In *C. lanceolata*, soil organic matter, total nitrogen, and most enzyme activities exhibited a “decrease-then-increase” pattern, reaching minima at Class III. In *Q. fabri*, these parameters generally increased with diameter class, peaking at Classes IV–V. Forest type, diameter class, and their interaction significantly influenced soil pH, total nitrogen, and enzyme activities. PCA identified diameter class as a primary driver of soil differentiation, with distinct shifts at Class III for *C. lanceolata* and Class I for *Q. fabri*. The interaction between diameter class and forest type affected SQI (*p* < 0.01): *Q. fabri* had higher SQI than *C. lanceolata* at Class V, whereas *C. lanceolata* had higher SQI than *Q. fabri* at Class I. Random forest models quantified biomass importance for SQI (test *R*^2^ > 0.75): aboveground biomass contributed more in *C. lanceolata* (feature weight = 0.57) and belowground biomass in *Q. fabri* (feature weight = 0.52). These findings reveal that tree–soil feedback are mediated by both ontogenetic stage and species identity, providing a mechanistic framework for diameter- and species-specific management in subtropical forest restoration.

## Figures and Tables

**Table 1:** Descriptive statistics of forests in different classes.

Forests Type	Size Class	Number of Plants	Factor	Minimum	Maximum	Mean	Median	Standard Deviation	Coefficient of Variable (%)
*C. lanceolata*	I	25	DBH (cm)	1.98	2.49	2.08	2.20	0.35	16.83
			TH (m)	1.09	2.31	1.69	2.10	0.61	36.09
			AB (kg)	0.38	0.96	0.63	0.78	0.14	22.02
			BB (kg)	0.05	0.18	0.12	0.15	0.08	63.62
			TB (kg)	0.43	1.15	0.75	0.93	0.20	26.92
	II	30	DBH (cm)	4.01	4.71	4.37	4.50	0.30	6.86
			TH (m)	3.62	4.81	4.39	4.00	0.56	12.76
			AB (kg)	2.59	2.97	2.73	2.70	0.21	8.19
			BB (kg)	0.50	0.65	0.53	0.58	0.09	16.16
			TB (kg)	3.23	3.62	3.26	3.28	0.29	9.40
	III	42	DBH (cm)	5.20	7.31	6.42	6.50	0.79	12.31
			TH (m)	4.50	6.91	5.28	5.50	2.11	39.96
			AB (kg)	1.06	10.67	5.88	7.90	2.86	48.65
			BB (kg)	0.68	2.95	1.54	1.80	0.67	43.48
			TB (kg)	2.53	13.62	7.42	9.70	2.98	40.18
	IV	15	DBH (cm)	7.50	14.90	12.28	10.30	2.13	17.35
			TH (m)	5.10	14.20	8.37	11.50	2.26	27.00
			AB (kg)	9.31	55.07	31.59	43.60	12.24	38.74
			BB (kg)	2.24	12.28	7.86	9.80	2.88	36.68
			TB (kg)	11.55	67.35	39.45	53.40	14.95	37.89
	V	8	DBH (cm)	15.00	24.30	17.81	18.10	2.19	12.30
			TH (m)	7.10	17.50	12.48	14.20	2.77	22.20
			AB (kg)	46.80	153.03	77.54	123.10	25.31	32.64
			BB (kg)	11.51	38.27	18.18	22.10	5.70	31.37
			TB (kg)	58.32	191.29	95.73	145.20	30.78	32.15
	total	120	DBH (cm)	1.98	24.30	12.89	14.25	5.24	36.77
			TH (m)	1.09	17.50	9.08	9.25	3.90	42.14
			AB (kg)	0.38	153.03	44.01	25.29	32.43	128.24
			BB (kg)	0.05	38.27	10.54	7.66	7.45	97.37
			TB (kg)	0.43	191.29	54.55	32.95	39.88	122.76
*Q. fabri*	I	15	DBH (cm)	1.10	2.40	1.92	2.10	0.41	21.35
			TH (m)	1.50	2.90	2.14	2.05	0.45	21.03
			AB (kg)	0.38	1.05	0.67	0.70	0.19	28.58
			BB (kg)	0.08	0.49	0.30	0.25	0.14	47.62
			TB (kg)	0.46	1.55	0.97	1.06	0.33	33.66
	II	28	DBH (cm)	2.60	4.80	3.93	3.95	0.71	18.07
			TH (m)	2.90	4.20	3.59	3.55	0.39	10.86
			AB (kg)	1.21	3.32	2.31	1.98	1.16	50.40
			BB (kg)	0.58	2.93	1.78	1.77	0.89	49.79
			TB (kg)	1.80	6.25	4.10	3.75	1.98	48.33
	III	30	DBH (cm)	5.10	7.40	6.23	6.25	0.72	11.56
			TH (m)	4.90	8.10	6.60	6.90	0.97	14.70
			AB (kg)	7.96	20.32	14.49	16.89	3.36	23.21
			BB (kg)	3.13	7.55	4.92	4.12	1.24	25.29
			TB (kg)	14.20	27.08	19.41	21.01	4.48	23.10
	IV	31	DBH (cm)	7.90	14.90	10.53	10.30	1.95	18.52
			TH (m)	6.80	11.80	9.09	8.80	1.19	13.09
			AB (kg)	24.67	91.45	48.10	42.29	18.09	37.61
			BB (kg)	7.14	29.01	14.59	13.50	5.86	40.15
			TB (kg)	31.80	120.47	62.69	55.78	23.78	37.93
	V	16	DBH (cm)	17.60	22.30	20.37	20.10	1.67	8.20
			TH (m)	11.20	15.60	13.83	14.20	1.73	12.51
			AB (kg)	167.52	272.06	213.49	207.29	30.36	14.22
			BB (kg)	40.21	71.38	54.38	53.74	9.26	17.04
			TB (kg)	207.73	343.43	267.87	261.03	38.92	14.53
	total	120	DBH (cm)	1.10	22.30	7.77	5.85	5.64	96.39
			TH (m)	1.50	15.60	6.66	6.95	3.73	53.64
			AB (kg)	0.38	272.06	41.14	13.59	67.38	495.80
			BB (kg)	0.08	71.38	11.82	4.32	17.53	405.71
			TB (kg)	0.46	343.43	52.96	17.91	84.91	473.26

Note: DBH represents the diameter at breast height of trees; TH represents tree height; AB represents aboveground biomass; BB represents belowground biomass; and TB represents total biomass, the sum of aboveground biomass and belowground biomass. *Cunninghamia lanceolata* (Lamb.) Hook. (*C. lanceolata*) and *Quercus fabri* Hance (*Q. fabri*) were the two tree species investigated in this study.

**Table 2:** Principal component analysis results, factor loadings, communal variances, and indicator weights for *Cunninghamia lanceolata* (Lamb.) Hook. forests.

Indicator	PC1	PC2	PC3	PC4	PC5	Common Factor Variance	Weights
Soil Bulk Density	−0.51	−0.34	−0.48	0.02	0.30	0.70	6.26
Non-capillary Porosity	0.13	0.60	0.51	0.53	−0.20	0.95	6.68
Capillary Porosity	−0.37	0.45	0.51	0.47	−0.06	0.83	6.72
pH	0.76	0.34	−0.33	0.06	−0.04	0.80	6.34
Soil Organic Matter	−0.05	0.77	−0.30	−0.05	0.19	0.72	5.17
Total Nitrogen	0.46	0.55	0.04	−0.17	0.60	0.90	6.38
Total Phosphorus	−0.40	0.10	0.46	0.28	0.58	0.80	5.87
Available Nitrogen	0.69	0.40	−0.23	0.08	0.23	0.74	6.43
Dissolved Organic Carbon	0.65	−0.40	0.31	0.12	0.11	0.70	6.34
Available Phosphorus	0.32	−0.69	−0.23	0.49	0.04	0.87	6.54
Dehydrogenase	−0.10	0.70	−0.38	−0.02	−0.21	0.68	5.33
Peroxidase	0.84	−0.34	−0.15	0.19	−0.05	0.88	6.43
Urease	0.34	0.82	0.17	−0.18	−0.16	0.87	6.53
Sucrase	0.62	−0.06	0.57	−0.34	−0.08	0.83	6.08
Acid Phosphatase	0.39	0.23	−0.70	0.36	−0.04	0.82	6.15
Nitrate Reductase	0.82	−0.35	0.31	−0.08	0.08	0.90	6.75
Eigen values	4.40	3.93	2.45	1.19	1.02		
Variance contribution	27.49	24.53	15.34	7.44	6.40		
Cumulative variance Contribution	27.49	52.02	67.36	74.80	81.20		

Note: PC1–PC5 represent the loadings of soil factors on the first five principal component axes.

**Table 3:** Principal component analysis results, factor loadings, communal variances, and indicator weights for *Quercus fabri* Hance forests.

Indicator	PC1	PC2	PC3	PC4	PC5	PC6	Common Factor Variance	Weights
Soil Bulk Density	0.59	−0.49	−0.41	0.18	−0.12	0.17	0.83	6.93
Non-capillary Porosity	−0.20	−0.35	0.55	−0.03	0.40	0.27	0.70	5.46
Capillary Porosity	0.52	0.04	0.20	−0.54	0.22	0.40	0.82	6.15
pH	−0.65	0.55	−0.11	0.17	0.32	0.16	0.89	6.95
Soil Organic Matter	0.62	0.01	0.58	0.08	−0.07	−0.24	0.79	5.74
Total Nitrogen	−0.01	0.77	−0.33	0.17	0.02	−0.31	0.83	4.83
Total Phosphorus	0.51	−0.29	0.38	0.38	0.04	−0.24	0.70	6.30
Available Nitrogen	0.43	−0.44	−0.31	0.46	0.40	−0.07	0.85	6.86
Dissolved Organic Carbon	0.51	0.38	0.40	0.47	−0.21	0.02	0.83	6.81
Available Phosphorus	0.19	0.68	0.05	0.30	−0.12	0.53	0.88	5.64
Dehydrogenase	0.63	0.25	0.04	0.17	0.44	0.02	0.68	5.61
Peroxidase	0.71	0.14	−0.06	−0.43	−0.40	−0.11	0.88	6.47
Urease	0.42	0.25	−0.33	−0.34	0.48	−0.34	0.81	6.73
Sucrase	0.57	−0.18	−0.51	0.21	−0.13	0.21	0.73	6.30
Acid Phosphatase	0.84	0.30	0.29	−0.15	0.09	−0.09	0.92	6.89
Nitrate Reductase	0.89	0.06	−0.28	−0.12	0.04	0.22	0.94	6.34
Eigen values	5.12	2.42	1.90	1.46	1.15	1.02		
Variance contribution	32.02	15.10	11.88	9.13	7.18	6.36		
Cumulative variance Contribution	32.02	47.13	59.01	68.14	75.32	81.68		

Note: PC1–PC6 represent the loadings of soil factors on the first six principal component axes.

**Table 4:** Binary allometric parameters for aboveground biomass estimation of *Cunninghamia lanceolata (Lamb.)* Hook. and *Quercus fabri* Hance in Hunan Province.

Forests Type	Diameter at Breast Height Range	Parameter Types	a_0_	a_1_	a_2_
*C. lanceolata*	DBH ≥ 5.0 cm	Binary	0.06539	2.01735	0.49425
*Q. fabri*		Binary	0.13188	1.82892	0.71119
*C. lanceolata*	DBH < 5.0 cm	Binary	0.19071	1.35226	0.49425
*Q. fabri*		Binary	0.15444	1.73082	0.71119

**Table 5:** Binary allometric parameters for belowground biomass estimation of *Cunninghamia lanceolata (Lamb.)* Hook. and *Quercus fabri* Hance in Hunan Province.

Forests Type	Diameter at Breast Height Range	Parameter Types	b_0_	b_1_	b_2_
*C. lanceolata*	DBH ≥ 5.0 cm	Binary	0.016385	2.52941	−0.11744
*Q. fabri*		Binary	0.114600	2.09235	−0.05245
*C. lanceolata*	DBH < 5.0 cm	Binary	0.034655	2.06398	−0.11744
*Q. fabri*		Binary	0.053436	2.56640	−0.05245

## Data Availability

The original contributions presented in this study are included in the article/Supplementary Material. Further inquiries can be directed to the corresponding author.

## Supplementary Materials

The following supporting information can be downloaded at: https://www.mdpi.com/article/10.3390/plants15030402/s1, Figure S1. *C. lanceolata* plot; Figure S2. *Q. fabri* plots; Figure S3. Site Description; Table S1. Distribution characteristics of *C. lanceolata* and *Q. fabri* in different classes; Table S2. Results of one-way ANOVA for soil physical and chemical properties across diameter classes in *C. lanceolata* and *Q. fabri* forests. Table S3. Results of one-way ANOVA for oil enzyme activities across diameter classes in *C. lanceolata* and *Q. fabri* forests. Table S4. Results of one-way ANOVA for soil nutrient indicators across diameter classes in *C. lanceolata* and *Q. fabri* forests.
