# Supplementary material for: Diameter Class-Dependent Species-Specific Tree–Soil Feedback Linked to Soil Quality Between Cunninghamia lanceolata (Lamb.) Hook. and Quercus fabri Hance in Subtropical Forests
Source: Plants (Basel). 2026 Jan 28;15(3):402. doi: 10.3390/plants15030402 (PMC12899702; doi:10.3390/plants15030402)
Supplement: Supplementary file 1 [file plants-15-00402-s001.zip › Supplementary Tables.pdf]

Table S1 Distribution characteristics of *C. lanceolata* and *Q. fabri* in different classes

Size Class	Number of plants	
	C. lanceolata	*Q. fabri*
Ⅰ	28	16
Ⅱ	34	29
Ⅲ	47	31
Ⅳ	17	32
Ⅴ	9	17
total	135	125

Note: The Table illustrates the distribution of the number of *C. lanceolata and Q. fabri* across different size classes.

Table S2. Results of one-way ANOVA for soil physical and chemical properties across diameter classes in *C. lanceolata* and *Q. fabri* forests.

Forests type	Soil Physical and Chemical Properties Indicators	Ⅰ	Ⅱ	Ⅲ	Ⅳ	Ⅴ
C. lanceolata	Soil bulk density	aA	aA	aA	aA	aA
Q. fabri		cB	bcA	aA	abA	bcA
C. lanceolata	Soil pH	abB	aA	cB	bA	aA
Q. fabri		aA	aA	bA	cA	abA
C. lanceolata	Non-capillary porosity	aA	aA	aA	aA	aA
Q. fabri		aA	aA	bA	cA	abA
C. lanceolata	Capillary porosity	aA	aB	aA	aB	aB
Q. fabri		bA	aA	abA	aA	aA

Note: Within species, different lowercase letters indicate significant differences among DBH classes ($P < 0.05$). Between species at the same DBH class, different uppercase letters indicate significant differences ($P < 0.05$).

Table S3. Results of one-way ANOVA for oil enzyme activities across diameter classes in *C. lanceolata* and *Q. fabri* forests.

Forests type	Soil Enzyme Indicators	I	II	III	Ⅳ	V
C. lanceolata	Soil peroxidase activity	bA	aA	cB	bcB	aA
Q. fabri		bA	bB	aA	aA	aA
C. lanceolata	Soil urease activity	aA	aB	bB	aB	abB
Q. fabri		aA	aA	aA	aA	aA
C. lanceolata	Soil dehydrogenase activity	bA	aA	aA	abA	cA
Q. fabri		bB	abB	aB	abB	aB
C. lanceolata	Soil sucrase activity	aB	abB	bB	bcB	abB
Q. fabri		bA	abA	aA	bcA	abA
C. lanceolata	Soil acid phosphatase	aB	aB	bB	aB	aB
Q. fabri		dA	cA	cA	bA	aA
C. lanceolata	Soil nitrate reductase	aA	aA	cB	cB	bB
Q. fabri		bB	aA	aA	aA	aA

Note: Within species, different lowercase letters indicate significant differences among DBH classes ($P < 0.05$). Between species at the same DBH class, different uppercase letters indicate significant differences ($P < 0.05$).

Table S4. Results of one-way ANOVA for soil nutrient indicators across diameter classes in *C. lanceolata* and *Q. fabri* forests.

Forests type	Soil Nutrient Indicators	Ⅰ	Ⅱ	Ⅲ	Ⅳ	Ⅴ
C. lanceolata	Soil organic matter level	bA	aA	cB	bcB	aA
Q. fabri		bA	bB	aA	aA	aA
C. lanceolata	Total nitrogen level	aA	aB	bB	aB	abB
Q. fabri		aA	aA	aA	aA	aA
C. lanceolata	Total phosphorus level	bA	aA	aA	abA	cA
Q. fabri		bB	abB	aB	abB	aB
C. lanceolata	Dissolved organic carbon	aB	abB	bB	bcB	abB
Q. fabri		bA	abA	aA	bcA	abA
C. lanceolata	Available nitrogen level	aB	aB	bB	aB	aB
Q. fabri		dA	cA	cA	bA	aA
C. lanceolata	Available phosphorus	aA	aA	cB	cB	bB
Q. fabri		bB	aA	aA	aA	aA

Note: Within species, different lowercase letters indicate significant differences among DBH classes ($P < 0.05$). Between species at the same DBH class, different uppercase letters indicate significant differences ($P < 0.05$).
