# Supplementary figures and images for: Diameter Class-Dependent Species-Specific Tree–Soil Feedback Linked to Soil Quality Between Cunninghamia lanceolata (Lamb.) Hook. and Quercus fabri Hance in Subtropical Forests
Source: Plants (Basel). 2026 Jan 28;15(3):402. doi: 10.3390/plants15030402 (PMC12899702; doi:10.3390/plants15030402)

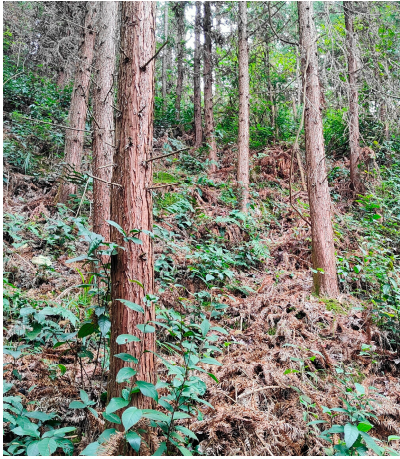

Fig. S1 *C. lanceolata* plot

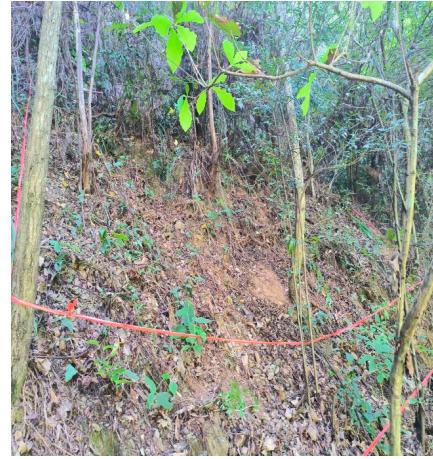

Fig. S2 *Q. fabri* plots

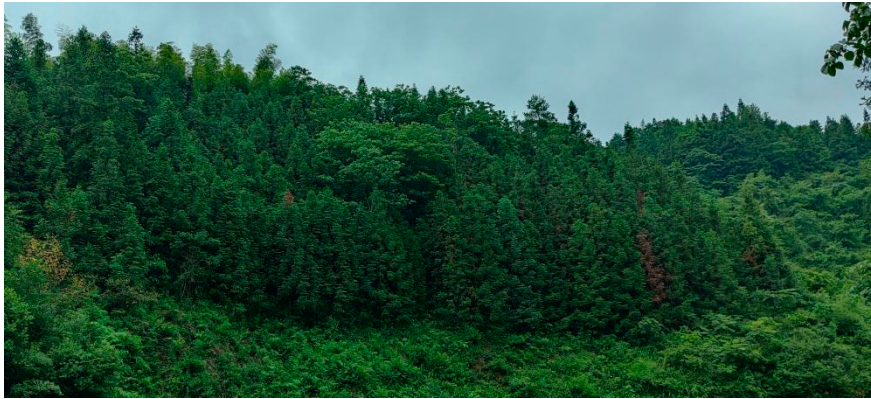

Fig. S3 Site Description

Supplement: Supplementary file 1 [file plants-15-00402-s001.zip › Supplementary Figures.pdf]
